# Functional confirmation of *PLAG1* as the candidate causative gene underlying major pleiotropic effects on body weight and milk characteristics

**DOI:** 10.1038/srep44793

**Published:** 2017-03-21

**Authors:** Tania Fink, Kathryn Tiplady, Thomas Lopdell, Thomas Johnson, Russell G. Snell, Richard J. Spelman, Stephen R. Davis, Mathew D. Littlejohn

**Affiliations:** 1 School of Biological Sciences, University of Auckland, Auckland, New Zealand; 2 Livestock Improvement Corporation, Hamilton, New Zealand

## Abstract

A major pleiotropic quantitative trait locus (QTL) located at ~25 Mbp on bovine chromosome 14 affects a myriad of growth and developmental traits in *Bos taurus* and *indicus* breeds. These QTL have been attributed to two functional variants in the bidirectional promoter of *PLAG1* and *CHCHD7*. Although *PLAG1* is a good candidate for mediating these effects, its role remains uncertain given that these variants are also associated with expression of five additional genes at the broader locus. In the current study, we conducted expression QTL (eQTL) mapping of this region using a large, high depth mammary RNAseq dataset representing 375 lactating cows. Here we show that of the seven previously implicated genes, only *PLAG1* and *LYN* are differentially expressed by QTL genotype, and only *PLAG1* bears the same association signature of the growth and body weight QTLs. For the first time, we also report significant association of *PLAG1* genotype with milk production traits, including milk fat, volume, and protein yield. Collectively, these data strongly suggest *PLAG1* as the causative gene underlying this diverse range of traits, and demonstrate new effects for the locus on lactation phenotypes.

Stature and body weight represent economically important traits in cattle. In beef animals, maximizing growth and development holds obvious importance for meat production. Despite modest positive correlations of body size with milk volume, protein, and fat yield^1^, smaller or larger animals may be desirable in dairy farming contexts, depending on management considerations. Stature and body weight are both highly heritable^2^, and large scale genetic studies have identified chromosomal regions impacting these traits in both *Bos taurus* and *indicus* species. Of these, a QTL on chromosome 14 with a major effect on stature and body weight was reported by Karim *et al*. in 2011^3^, and has since been observed in many independent populations^4^,^5^,^6^,^7^.

In a detailed analysis of this chromosome 14 locus^3^, fine mapping yielded 13 candidate polymorphisms as potentially underlying the QTL, none of which mapped to protein coding sequences. Further functional and genetic analysis reduced the number of candidate variants to two polymorphisms in the bidirectional promoter of the *PLAG1* and *CHCHD7* genes; rs209821678, a (CCG) repeat of 9 or 11 copies and rs210030313 an A to G nucleotide substitution^3^. In this analysis, both variants were associated with foetal expression of seven of the nine genes within a ~780 kilobase (kb) interval representing the stature QTL. Of these genes, *PLAG1, RPS20*, and *SDR16C5* were plausible biological candidates for these effects, with demonstrated roles in growth and oncogenesis^8^,^9^,^10^. In particular, *PLAG1* represented an obvious candidate given *plag1* knockout mice suffer from slow growth rates and dwarfism^11^. This gene encodes a transcription factor that regulates several growth factors including IGF2^10^,^12^, a key modulator of growth in both dogs and humans^13^,^14^. Despite these observations, given the differential expression of multiple genes at this locus, the causative status of these genes remains to be resolved.

In the current study, we have used a mammary RNAseq dataset (N = 375) to perform expression QTL (eQTL) mapping in a 2 Mbp interval encompassing the previously implicated chromosome 14 locus. We report genetic effects for a subset of the nine genes of interest, and further report investigation of milk composition and body weight effects in a separate population (N = 39,391) of lactating cows.

## Results

### eQTL analysis of the chromosome 14 locus

To quantify the expression levels of the nine genes of interest at the chromosome 14 body weight locus, high-depth, mammary RNAseq data from 375 lactating cows was assessed. Five of these nine genes were appreciably expressed (see Methods), consisting of *CHCHD7, IMPAD1, LYN, PLAG1* and *RPS20*. Of these, *RPS20* was by far the most abundantly expressed (109.6 FPKM), and *PLAG1* was relatively lowly expressed (0.8 FPKM).

**Figure 1:**
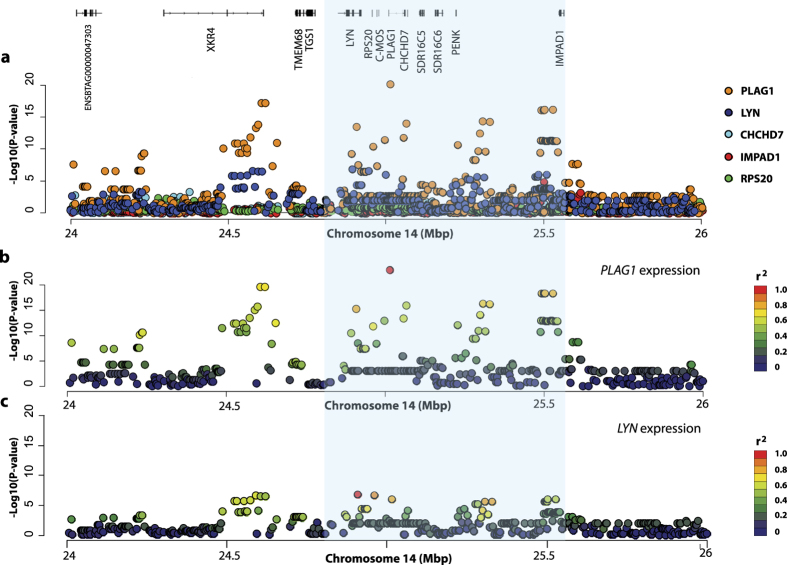
Expression QTL analysis at the chromosome 14 locus. (**a**) shows layered Manhattan plots for the five nominally expressed genes at the previously reported, ~780 kb body weight locus (blue-shaded area) in RNA-sequenced animals. The X-axis shows bp position on chromosome 14, the Y-axis shows −log10 P-values of marker association. The location and structure of 14 genes mapping to the broader, 2 Mbp interval are shown at the top of field. (**b**) indicates the marker association of the 432 SNPs with *PLAG1* expression. (**c**) indicates marker association with *LYN* expression. The top gene expression-associated SNP is coloured red in (**b**) and **c**, with other variants coloured according to their linkage disequilibrium relationship with these SNPs.

Association analysis of these five genes was then conducted to test for eQTL effects. In these analyses, transformed read counts for each gene were tested in conjunction with 432 Illumina BovineHD SNPs located in a 2 megabase (Mbp) interval encompassing the previously published QTL interval. This region was centred on the BovineHD panel variant rs109815800 (Chr14:25015640, UMD3.1 genome build), selected as a tag-SNP of the two putative causative variants, since this SNP has been shown to be in complete LD with these polymorphisms in New Zealand Holstein-Friesians, Jerseys and their crosses^4^. Restricted maximum likelihood analysis using pedigree-based mixed models revealed significant eQTLs for three of five expressed genes in the QTL interval (Fig. 1a, Table 1). However, only *PLAG1* and *LYN* were significantly differentially expressed by rs109815800 genotype. For *PLAG1,* rs109815800 was the most significant SNP (P = 1.33 × 10^−23^; Fig. 1a and b; Table 2), which was 17 orders of magnitude more significant than the association with *LYN* expression (P = 1.15 × 10^−6^; Fig. 1a and c Table 2). Notably, the genetic signature of the *LYN* eQTL appeared to differ from the *PLAG1* eQTL, with a different lead SNP (P = 1.71 × 10^−7^; rs109116062; chr14:24909247; Fig. 1a,b and c; Table 1), and with the rs109815800 variant appearing lower in the association rankings for *LYN*. The rs109815800 SNP explained 46.6% of the genetic variance and 32.6% of the phenotypic variance in *PLAG1* expression, and 45.2% and 9.6% respectively for expression of *LYN* (Table 2). This contrasted with 61.6% and 9.9% respectively for the lead *LYN* SNP rs109116062, again suggesting that the *PLAG1* and *LYN* eQTLs might be being driven by different or overlapping (i.e. functionally independent, yet genetically linked) effects. The frequency of the body weight-increasing ‘G’ allele was 0.68 in the mostly Holstein-Friesian RNAseq population.

### Analysis of body weight and milk composition effects

Having observed differential expression of *PLAG1* and *LYN* by rs109815800 genotype, we wondered whether these eQTL might have phenotypic consequences in the mammary gland. To answer this question, we used a population of 39,391 lactating cows for which both milk production and body weight data were available, using association models similar to those used for eQTL analysis (see Methods). Given the major milk production effects attributed to another (albeit distant) chromosome 14 mutation, the *DGAT1* K232A variant^15^, these models also incorporated imputed *DGAT1* K232A genotypes as a fixed effect (see Methods). Targeting the same interval of BovineHD SNPs used previously, analysis of body weight confirmed a very large effect at this locus, with the causative mutation tag-SNP rs109815800 the most significant variant (P < 2.2 × 10^−308^, Table 3). This SNP accounted for 32.8% of the genetic variance and 15.8% of the phenotypic variance in body weight for this population, and notably, the body weight-increasing ‘G’ allele was the same allele associated with increased *PLAG1* gene expression.

**Figure 2:**
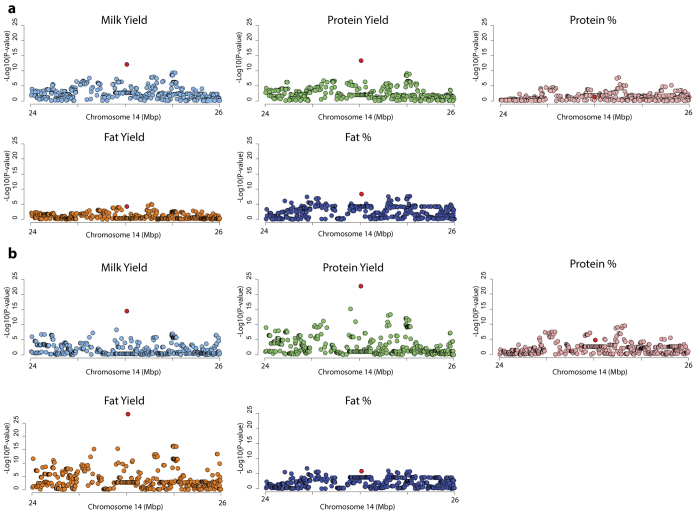
Milk composition QTLs at the chromosome 14 locus. (**a**) represents the Manhattan plots for the five milk composition and yield phenotypes, with the X-axis showing Mbp position on chromosome 14, and the Y-axis showing −log10 P-values of marker association. (**b**) represents similar plots where animal body weight has been fitted as an additional covariate in otherwise identical association models. The rs109815800 SNP is coloured red in both (**a**) and (**b**).

Next, we conducted association mapping of milk volume, milk fat and protein yield, and milk fat and protein percentage traits. The frequency of the body weight-increasing ‘G’ allele was 0.46 in this population of mixed breed cattle. Significant effects were observed for all traits except milk protein percentage (Fig. 2a; Table 3), where the body weight increasing rs109815800 ‘G’ allele was associated with increased milk volume and milk protein and fat yield, and decreased milk fat percentage. Given the magnitude of association of rs109815800 genotype with body weight, we reasoned that some of these associations might be due to differences in animal (and thus mammary) size^16^. To attempt to differentiate these effects from those that might be impacting secretory pathways and/or energy utilisation irrespective of size, we conducted an alternative analysis that fitted animal body weight as an additional covariate in the association models (see Methods). Interestingly, rs109815800 showed significant associations for all milk traits using these models (Table 4), including a highly significant reduction in milk fat yield, and decreases in milk volume and protein yield in animals carrying the *PLAG1* high-expression ‘G’ allele. The sign of effect was reversed for milk volume in these body weight-adjusted models, suggesting that the increased size of animals carrying rs109815800 ‘G’ alleles was indeed playing a role in the milk production effects observed at this locus.

Since the chromosome 14 body weight QTL minor allele is known to differ between Holstein-Friesian and Jersey breeds^3^, and that this might be expected to lead to population stratification and confound analyses in a mixed breed population, we also conducted analysis of body weight and lactation traits in purebred animals. These analyses used purebred-segregated (16/16ths breed proportions) Holstein-Friesian (N = 8086) and Jersey (N = 4322) subpopulations (see Methods), and confirmed significant effects for a subset of the traits (Tables 3 and 4). Although body weight was the only significantly associated phenotype in Jersey animals, statistical power was limited in this population due to a low MAF (0.02). With the exception of milk fat yield in the body weight-unadjusted models (Table 3), the direction of effects was otherwise identical across all populations and traits (including significant and non-significant associations alike). These observations suggested that association-confounding by breed was unlikely to be a major issue in these analyses, further supporting the role of *PLAG1* as a gene with pleiotropic impacts on these traits.

## Discussion

We report a strong mammary eQTL for *PLAG1* which bears the same genetic signal underpinning the body weight and developmental QTLs reported for this locus. To our knowledge, these data represent the first functional confirmation of this expression-based effect. While contrary to previous analysis in foetal tissues showing *cis* eQTL for multiple genes^3^, the current analysis suggests *PLAG1* alone as responsible for these effects. We additionally report new associations of *PLAG1* genotype with milk composition and yield phenotypes, adding lactation effects to the long list of physiological traits that are impacted by this locus.

Karim *et al*. (2011) first reported the presence of eQTLs underpinning the bovine stature locus on chromosome 14. They performed quantitative PCR using foetal brain, bone, muscle and liver samples representing 79 individuals and found significant associations with the expression of *RPS20, MOS, PLAG1, CHCHD7, SDR16C5, SDR16C6* and *PENK* genes. While it is possible that all genes in this interval are affected by a single control element in foetal tissues, it is also plausible that these associations were due to the genotype tagging multiple independent eQTL, given that association testing was restricted to analysis of a single variant. Critically, of the seven candidates above, only *PLAG1* was significantly differentially expressed by body weight QTL genotype in mammary tissue, with both eQTL and physiological trait QTL sharing the same top-associated SNP. Although we also observe an association with *LYN* expression, the rank order of associated SNPs suggests these QTLs may be driven by a different genetic element, and given that no eQTL was reported in foetal tissues, *LYN* can likely be discounted as a candidate for the stature and body weight effects. Taken together, these observations provide further evidence for *PLAG1*, likely under regulatory control of the rs209821678 and/or rs210030313 variants proposed be Karim *et al*. (2011), as the causative gene responsible for these effects. Since mammary tissue was used for eQTL mapping in the current study, this hypothesis assumes a shared regulatory architecture between this tissue and tissues of more direct relevance to growth and development processes. As such, an alternative hypothesis proposes the lactation effects being driven by *PLAG1*, and the previously reported stature and body weight QTLs being underpinned by one of the other candidate genes highlighted by Karim *et al*. (2011). Although technically conceivable, we contend the simplest and most plausible hypothesis is one that sees a single, major-effect pleiotropic *PLAG1* eQTL underpinning all physiological effects.

Given observation of the mammary eQTL effects, and the many other pleiotropic observations at this locus, we wondered whether differential expression of *PLAG1* might impact lactation traits directly. We analysed milk volume, milk fat percentage and yield, and milk protein percentage and yield, and found highly significant associations of rs109815800 with all traits except protein percentage. The body weight-increasing ‘G’ allele was associated with increased milk volume, fat, and protein yield, and decreased fat percentage. This opposing sign of effect between component yields and percentages could reflect these animals producing a higher volume of milk relative to the increases seen in the milk components, resulting in milk that is marginally more dilute. This phenomenon of milk component and yield effects being co-ordinately impacted is something that we^17^,^18^, and others^19^, have observed for other major QTL previously.

However, given the profound impact of this locus on animal stature and body weight, and the fact that larger cattle produce more milk^20^, we reasoned that volume effects might reflect differences in mammary size and capacity. As expected, adjusting for animal body weight in the association models revealed that this initial association was likely driven by differences in animal size, and notably, the sign of the SNP effect was reversed in this model. This observation was apparent for milk volume, protein, and fat yield traits, where the ‘G’ allele was associated with *decreased* yields, suggesting an efficiency advantage to the alternate allele. The apparent reduction in milk fat yield in animals carrying the allele normally associated with increased body weight was the most significant effect in these models and is of further note, since effects on reduced intramuscular fat and fat deposition have also been reported for this allele^7^. The mobilisation of body fat reserves to support the greater energy requirements of the lactating mammary gland is well described^21^,^22^. We speculate that this reduction in milk fat may be due to differential energy utilisation between genotypes, whereby energy normally partitioned into subcutaneous fat or milk triglyceride synthesis shifts to a balance favouring increased lean tissue mass. Additional physiological indicators of energy balance and lipolysis could be examined in animals of contrasting *PLAG1* genotype to further test this hypothesis.

It is interesting to contemplate what mammary-specific pathways may be involved in the lactation effects proposed in our study. Two well-demonstrated targets of *PLAG1* signalling include molecules of the IGF2 and WNT pathways^10^,^23^, with the former speculated as the underlying mechanism of the growth and body weight effects attributed to this QTL^3^,^24^. Transgenic mouse lines engineered to overexpress *plag1* in mammary tissue show differential expression of IGF2 and WNT signalling genes^25^, with mammary hyperplasia and development of adenomyoepitheliomas the primary phenotypes of these models. There is limited data to suggest IGF2 may increase milk synthesis in the lactating mammary gland^26^, though the role of the hormone in mammary development and involution is clearly demonstrated^27^,^28^. Likewise, WNT signalling is proposed to play important roles in the development and differentiation of the mammary gland during pregnancy^29^, and assuming the involvement of *PLAG1* in these pathways is relevant outside of a tumorigenic context, the effects demonstrated might derive from morphological differences between animals of different QTL genotype. It is also possible that the milk composition and yield effects may reflect secondary impacts of the QTL deriving from effects in other tissues. Given that *PLAG1* is expressed during lactation and the eQTL is observed during this period, however, another appealing mechanism is one that acts through some as yet unidentified factors with direct, modulatory roles on milk synthesis and secretion.

## Conclusions

In summary, we describe a strong mammary eQTL for *PLAG1* that bears the same genetic signal underpinning the previously described body weight and developmental effects at this locus. We additionally report new associations of *PLAG1* genotype with milk composition and yield phenotypes. These data provide the first functional validation of an eQTL-mediated mechanism underpinning these QTLs, and further expand the list of pleiotropic effects attributed to *PLAG1* in bovine species.

## Methods

### Ethics statement

All animal experiments were conducted in strict accordance with the rules and guidelines outlined in the New Zealand Animal Welfare Act 1999. For the mammary tissue biopsy experiment, samples were obtained in accordance with protocols approved by the Ruakura Animal Ethics Committee, Hamilton, New Zealand (approval AEC 12845). All other data were generated as part of routine commercial activities outside the scope of that requiring formal committee assessment and ethical approval (as defined by the above guidelines). No animals were sacrificed for this study.

### Primary data

Primary datasets consisting of relevant genotypes, and milk production and gene expression phenotypes have been deposited into the Dryad digital data repository^30^ (doi: 10.5061/dryad.r8251), and NCBI Short Read Archive (SRP075939).

### Animal populations

#### RNAseq biopsy animals

Animals used for RNAseq analysis comprised 375 mostly Holstein-Friesian NZ dairy cows, representing a subset of 406 sequenced animals described in detail previously^17^,^18^.

#### Milk production and body weight population

The animal population used for GWAS comprised 39,391 dairy cows, consisting of 8,086 Holstein-Friesians, 4,322 Jerseys, and 26,983 Holstein-Friesian x Jersey cross breeds, where Holstein-Friesians and Jerseys were considered pure with a breed proportion of 16/16ths. This population represents part of a larger phenotypic and genotypic database of animals used for evaluation of sire performance, similar to populations described previously^17^,^18^. Animals were also segregated by breed (as defined above) to assess within-breed effects and potential confounding impacts of population stratification. Slight differences in the animal numbers quoted for each analysis is a reflection of the quality filtering performed on each trait.

#### Milk composition and body weight assessment

Milk composition phenotypes were derived from first lactation herd test data. Concentrations of major milk components were measured using Fourier transform infrared spectroscopy as part of standard herd testing procedures as described in refs 17 and 18. These concentrations were adjusted using linear models with age at calving and stage of lactation as fixed effects and contemporary group as an absorbed/sparse fixed effect. Residuals from these models were used for subsequent association analyses. Body weight measurements were from two sources, either representing a weight where the animal walked over a scale or a weight derived from visual scoring carried out by certified assessors in accordance with published guidelines^31^. Body weight records were restricted to values measured on two year olds in their first lactation and were filtered to remove outliers. Individual estimates for each animal were derived by fitting a repeated measures model in ASReml-R and were used for subsequent association analyses. Fixed effects included in the model were method of measurement (scale weight/inspector weight), age at calving, and stage of lactation with contemporary group fitted as an absorbed/sparse fixed effect.

#### DNA extraction, high throughput genotyping, and genotype imputation

Genomic DNA extraction was conducted as previously described^17^,^18^. Briefly, DNA was extracted from either blood or ear-punch tissue or processed using Qiagen Biosprint kits (Qiagen) or a MagMax system (Life Technologies) by GeneMark (Hamilton, New Zealand), and GeneSeek (Lincoln, NE, USA), respectively. Genotyping was conducted by GeneSeek (Lincoln, NE, USA), using the Illumina BovineHD BeadChip or BovineSNP50 BeadChip (Illumina) platforms. For samples genotyped on BovineSNP50 chips, these were imputed to the BovineHD platform using Beagle software (Beagle v3.3.2)^32^ prior to association analysis, using methods similar to those described previously^17^,^18^. Briefly, for the small subset of RNAseq animals that had not been physically genotyped on the BovineHD Beadchip (N = 27 cows), imputation was performed on a genome-wide basis for 659,811 SNP using a reference population of 3,460 animals (with 46,805 SNPs overlapping between platforms). For the population of 39,391 cows used for milk composition and body weight analysis, a reference population of 3389 animals was used to impute 675,321 SNPs (with 46,621 SNPs overlapping between platforms). The *DGAT1* K232A variant was imputed from whole genome sequence data using a reference population of 556 animals, in an approach similar to that described previously^18^. Linkage disequilibrium statistics for all chromosome 14 SNPs used for association analysis are shown in Supplementary Table 1, calculated on the larger (N = 39,391) of the two animal populations.

### RNA sequencing

High-depth RNAseq was undertaken using mammary gland biopsies from lactating cows as described in detail previously^18^. Briefly, 21 of the 375 samples were collected in 2004 and 2012, and were sequenced by NZGL (Dunedin, New Zealand) using the Illumina HiSeq 2000 instrument. The remaining samples were collected in 2013 and 2014 and were sequenced by the Australian Genome Research Facility (AGRF; Melbourne, Australia) using the Illumina HiSeq 2000 instrument.

### RNA sequence informatics

RNA sequence reads were mapped to the UMD3.1 genome using Tophat2 (version 2.0.12)^33^ as previously described^18^. Cufflinks software (version 2.1.1)^34^ was used to quantify expressed transcripts, yielding fragments per kilobase of exon model per million mapped (FPKM) expression values. The genes in the ~780 kb region of interest on chromosome 14 were considered for downstream analysis if they had non-zero FPKM values in at least 75% of samples, and had a mean expression of 0.5 FPKM or greater.

To derive gene expression phenotypes suitable for eQTL analysis, the read counts from the nine genes in this interval were also processed using the variance-stabilising transformation (VST) method in DESeq (version 1.18)^35^. This transformation addresses issues of heteroscedasticity inherent in RNA-seq data, and normalises the count data to a form suitable for linear model analysis.

### Genetic association analysis

For the 2 Mbp interval of Illumina BovineHD SNPs encompassing the rs109815800 variant, associations with gene expression, body weight, and milk composition phenotypes were quantified using pedigree-based mixed models in ASReml-R^36^,^37^. The RNAseq analysis used a total of 432 SNPs, the body weight and milk composition analyses used a subset of 421 SNPs with the difference reflecting the impact of slightly different imputation and quality-filtering criteria applied between populations. Each SNP was fitted in a separate sire-maternal grandsire single-trait model with the SNP treated as a quantitative variable based on the number of copies of the alternative allele and variance components estimated in a restricted maximum-likelihood (REML) framework. Covariates for birth year, the proportions of NZ Holstein-Friesian ancestry, US Holstein-Friesian ancestry, Jersey ancestry and breed heterozygosity effects were also included in the models. The additive genetic variance for each SNP was calculated using 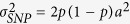, where *p* is the frequency of the highest frequency allele and 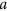 is the estimated allele substitution effect. Polygenic genetic variances were evaluated as 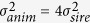 where 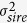 is the estimate of sire variance from the model. Total genetic variance was evaluated as 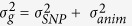 and phenotypic variance was evaluated as 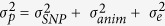 where 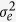 is the error variance. The proportion of phenotypic variance explained by each SNP for each phenotype was calculated as 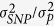 and the proportion of genetic variance explained by each SNP was calculated as 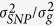.

For eQTL analysis, these models used VST-normalised read counts from the mapped RNA-seq data, representing the five nominally expressed genes in the QTL interval. These models also included a fixed effect for biopsy year to address batch variation between the different sequencing submissions. For milk composition and body weight analysis, models used the body weight and milk composition phenotypes described above. For the association analyses that considered body weight in the analysis, these models were conducted in the same way, with the addition of body weight as another covariate. Models also included a fixed effect to account for potentially confounding impacts of the *DGAT1* K232A mutation. This variant is known to have profound impacts on milk composition^15^, and despite being >23 Mbp away, long distance linkage disequilibrium might be anticipated to influence milk composition association results.

Associations were considered significant using an alpha value of 0.05 that incorporated Bonferroni corrections for multiple hypothesis testing across the different study populations. For eQTL analyses, a total of 2160 tests were conducted (five gene expression traits x 432 SNPs), yielding a nominal significance threshold of P = 2.31 × 10^−5^. For milk composition and body weight analyses, a nominal significance value of P = 1.51 × 10^−3^ was used (33 tests).

## Additional Information

**How to cite this article**: Fink, T. *et al*. Functional confirmation of *PLAG1* as the candidate causative gene underlying major pleiotropic effects on body weight and milk characteristics. *Sci. Rep.* **7**, 44793; doi: 10.1038/srep44793 (2017).

**Publisher's note:** Springer Nature remains neutral with regard to jurisdictional claims in published maps and institutional affiliations.

## Supplementary Material

Supplementary Information

## Figures and Tables

**Table 1:** Chromosome 14 body weight locus effect on gene expression.

Gene	Top SNP	Location on chr 14 (bp)	P-value
***PLAG1***	**rs109815800**	**25015640**	**1.33E-23**
***LYN***	**rs109116062**	**24909247**	**1.71E-07**
*CHCHD7*	rs42648880	24378496	4.33E-04
*RPS20*	rs134518689	24278284	0.008
***IMPAD1***	**rs110632518**	**25501417**	**1.69E-05**

For each of the five mammary-expressed genes in the 2 Mbp interval of interest, the top-associated SNP, its position and P-value of association is indicated. Significant effects are bolded; multiple testing significance threshold P = 2.31 × 10^−5^.

**Table 2:** Association between rs109815800 and the expression of genes at the chromosome 14 body weight locus (N = 375 animals).

Gene	Effect ± SE	Pheno var explained	Geno var explained	P-value
***PLAG1***	**−0.5293 (±0.0491)**	**32.59**	**46.59**	**1.33E-23**
***LYN***	**0.0787 (±0.0159)**	**9.61**	**45.22**	**1.15E-06**
*CHCHD7*	−0.0305 (±0.0165)	N/A	N/A	0.065
*RPS20*	−0.0179 (±0.0251)	N/A	N/A	0.476
*IMPAD1*	0.0059 (±0.0193)	N/A	N/A	0.760

Effect estimates are given with standard errors in units of VST read counts. For significant effects (bolded), the ‘Pheno var explained’ and ‘Geno var explained’ columns represent the percentage of phenotypic and genotypic variance accounted for by the rs109815800 SNP. P-values of genetic association are indicated in the right-most column. Significance threshold P = 2.31 × 10^−5^.

**Table 3:** Association between rs109815800 and milk composition traits and body weight in NZ dairy cows.

Phenotype	All animals					Holstein-Friesian			Jersey		
	N	Effect ± SE	Pheno var explained	Geno var explained	P-value	N	Effect ± SE	P-value	N	Effect ± SE	P-value
Milk volume	**39380**	**−0.0815 ± 0.0114**	**0.239**	**0.879**	**8.96E-13**	8082	−0.0841 ± 0.027	1.98E-03	4321	−0.094 ± 0.079	0.234
Fat %	**39391**	**0.0198 ± 0.0034**	**0.155**	**0.309**	**4.21E-09**	**8086**	**0.0401 ± 0.0067**	**2.48E-09**	4322	0.0134 ± 0.0296	0.650
Fat yield	**39374**	**−1.628 ± 0.404**	**0.076**	**0.345**	**5.52E-05**	8084	0.2218 ± 0.8828	0.802	4321	−3.552 ± 3.132	0.257
Protein %	39391	0.0028 ± 0.0017	N/A	N/A	0.098	8086	0.0123 ± 0.0035	3.84E-03	4322	0.0186 ± 0.0143	0.194
Protein yield	**39376**	**−2.293 ± 0.304**	**0.267**	**1.239**	**4.92E-14**	8082	−1.518 ± 0.709	0.032	4320	−2.081 ± 2.238	0.352
Body weight	**39391**	**−17.25 ± 0.27**	**15.80**	**32.78**	**<2.23E-308**	**8086**	**−15.72 ± 0.633**	**4.01E-131**	**4321**	**−19.997 ± 1.939**	**1.20E-24**

Association results for the rs109815800 SNP with milk composition and body weight phenotypes are shown for models fitted across all animals and when segregated to Holstein-Friesian and Jersey breeds. Effects are expressed for each ‘T’ allele relative to homozygous ‘G’ animals, displayed using units of grams for yield traits, litres for milk volume, and kilograms for body weight. For significant effects (bolded), ‘Pheno var explained’ and ‘Geno var explained’ columns represent the percentage of phenotypic and genotypic variance accounted for by the rs109815800 SNP. Significance is based on a Bonferroni-adjusted threshold of P = 1.51 × 10^−3^.

**Table 4:** Association between rs109815800 and milk composition traits conditioned on body weight in NZ dairy cows.

Phenotype	All animals					Holstein-Friesian			Jersey		
	N	Effect ± SE	Pheno var explained	Geno var explained	P-value	N	Effect ± SE	P-value	N	Effect ± SE	P-value
Milk volume	**39380**	**0.0918 ± 0.0116**	**0.322**	**1.226**	**3.17E-15**	8082	0.0808 ± 0.0274	3.17E-03	4321	0.086 ± 0.078	0.27
Fat %	**39391**	**0.017 ± 0.0035**	**0.115**	**0.23**	**1.49E-06**	**8086**	**0.0341 ± 0.007**	1.04E-06	4322	0.0127 ± 0.0299	0.671
Fat yield	**39374**	**4.615 ± 0.412**	**0.65**	**3.087**	**4.37E-29**	**8084**	**5.411 ± 0.891**	1.30E-09	4321	4.178 ± 3.08	0.175
Protein %	**39391**	**0.0078 ± 0.0018**	**0.091**	**0.165**	**1.78E-05**	**8086**	**0.0156 ± 0.0036**	1.50E-05	4322	0.0222 ± 0.0145	0.125
Protein yield	**39376**	**3.066 ± 0.307**	**0.516**	**2.614**	**2.11E-23**	**8082**	**3.311 ± 0.706**	2.76E-06	4320	3.586 ± 2.196	0.103

Association results for the rs109815800 SNP with milk composition phenotypes conditioned on body weight are shown for models fitted across all animals and when segregated to Holstein-Friesian and Jersey breeds. Effects are expressed for each ‘T’ allele relative to homozygous ‘G’ animals, displayed using units of grams for yield traits, and litres for milk volume. For significant effects (bolded), ‘Pheno var explained’ and ‘Geno var explained’ columns represent the percentage of phenotypic and genotypic variance accounted for by the rs109815800 SNP. Significance is based on a Bonferroni-adjusted threshold of P = 1.51 × 10^−3^.
