# Supplementary material for: Functional confirmation of PLAG1 as the candidate causative gene underlying major pleiotropic effects on body weight and milk characteristics
Source: Sci Rep. 2017 Mar 21;7:44793. doi: 10.1038/srep44793 (PMC5359603; doi:10.1038/srep44793)
Supplement: Supplementary Information [file srep44793-s1.pdf]

Supplementary Information from:

Functional confirmation of *PLAG1* as the candidate causative gene underlying major pleiotropic effects on body weight and milk characteristics

Tania Fink[2], Kathryn Tiplady[1], Thomas Lopdell[1,2], Thomas Johnson[2], Russell G Snell[2], Richard J Spelman[1,] Stephen R Davis[1], Mathew D Littlejohn*[1,2]

1. Livestock Improvement Corporation, Hamilton, New Zealand
2. School of Biological Sciences, University of Auckland, Auckland, New Zealand

Supplementary Table 1. Linkage disequilibrium statistics (R^2 values) between rs109815800 and DGAT1 K232A/BovineHD panel markers in chromosome 14 body weight locus

Marker	Position	R^2
rs109234250	1802265	0.063
rs109637592	24008839	0.141
rs109925810	24014579	0.465
rs110489692	24018803	0.162
rs136508017	24044381	0.328
rs42545204	24047418	0.164
rs41660107	24048952	0.328
rs109602517	24049812	0.328
rs109318512	24051093	0.328
rs109705035	24051987	0.150
rs109284285	24053137	0.328
rs110845339	24057354	0.328
rs42545192	24065280	0.163
rs133032517	24067610	0.212
rs109422239	24072137	0.212
rs110821373	24074220	0.212
rs42545182	24075714	0.162
rs110390285	24089516	0.070
rs109643003	24092123	0.212
rs136339222	24096532	0.132
rs42545165	24099094	0.162
rs134304712	24099719	0.132
rs137341544	24102024	0.132
rs109528593	24106396	0.212
rs110864751	24114365	0.433
rs42545145	24115422	0.054
rs42544420	24132456	0.321
rs42544424	24133627	0.217
rs42544430	24138878	0.217
rs109682353	24143265	0.321
rs41660101	24145838	0.321
rs110006364	24147525	0.433
rs42544418	24150127	0.217
rs42544401	24158787	0.219
rs42544357	24161697	0.050
rs42544377	24167861	0.217
rs42544383	24172479	0.323
rs42544386	24175600	0.324
rs42544392	24179150	0.207
rs42544395	24181858	0.324
rs42544396	24182406	0.207
rs42544400	24185058	0.324
rs42544356	24187772	0.324
rs42544349	24191959	0.324
rs42544348	24193383	0.324
rs134319614	24206232	0.023
rs109890494	24219041	0.435
rs42544336	24220070	0.000
rs110993288	24221657	0.435
rs109174538	24222338	0.434
rs109976467	24225369	0.435
rs108982003	24226206	0.434
rs110482368	24227327	0.434
rs134751608	24229059	0.501
rs110010333	24235712	0.493
rs109645403	24237304	0.493
rs42543230	24243733	0.323
rs133885118	24258275	0.006
rs137434020	24260937	0.006
rs136017102	24263980	0.027
rs43004834	24266960	0.000
rs41581840	24275232	0.018
rs109963694	24276214	0.124
rs134518389	24278284	0.036
rs135639509	24280431	0.006
rs136703875	24281870	0.006
rs134312033	24285339	0.002
rs133296385	24291712	0.028
rs43003348	24304427	0.037
rs43002526	24321232	0.183
rs137785718	24323400	0.102
rs110104035	24324094	0.103
rs110383563	24326513	0.021
rs134955677	24329536	0.096
rs43003344	24330594	0.268
rs134193404	24333423	0.084
rs135748649	24335922	0.084
rs132711884	24336953	0.084
rs109341693	24348047	0.029
rs135409116	24359161	0.272
rs42648898	24361242	0.290
rs41615249	24363276	0.024
rs29020689	24365162	0.316
rs134293561	24369510	0.269
rs135459952	24373031	0.269
rs133288868	24376195	0.269
rs42648880	24378496	0.016
rs136831935	24384496	0.201
rs135164902	24385879	0.125
rs42648868	24391175	0.051
rs135413008	24395527	0.138
rs137782768	24396836	0.137
rs133719195	24400605	0.138
rs137364314	24404982	0.138
rs133138223	24406302	0.137
rs42648925	24411455	0.067
rs42649744	24412493	0.067
rs42649760	24413758	0.140
rs109119025	24418370	0.060
rs42649767	24419295	0.083
rs42649771	24420840	0.122
rs109185321	24425758	0.104
rs110340643	24429310	0.047
rs110717761	24431237	0.104
rs136321755	24434190	0.051
rs42649775	24437778	0.187
rs42649776	24440797	0.054
rs109425569	24445514	0.073
rs110395333	24448641	0.073
rs109828753	24453615	0.055
rs42649778	24455791	0.084
rs110727287	24459302	0.055
rs42649780	24466047	0.187
rs135958550	24470245	0.187
rs110543321	24471148	0.187
rs42646633	24472819	0.111
rs42646635	24473841	0.156
rs42646636	24475213	0.156
rs42646638	24476256	0.156
rs132820259	24478336	0.156
rs42646648	24487011	0.352
rs42646660	24524205	0.463
rs132924262	24532336	0.467
rs42646685	24536549	0.314
rs42646677	24545053	0.314
rs135646716	24553162	0.464
rs42646691	24556301	0.359
rs42646700	24562756	0.313
rs42646702	24563237	0.352
rs42646708	24573257	0.466
rs42646720	24590812	0.479
rs134188138	24595318	0.031
rs42646723	24598515	0.477
rs41724398	24621142	0.667
rs133020056	24633076	0.238
rs41723523	24639618	0.049
rs41724332	24643266	0.413
rs109080115	24656389	0.597
rs135008823	24699409	0.104
rs136704276	24706121	0.099
rs109748092	24710609	0.328
rs108959399	24716826	0.328
rs110451945	24718647	0.328
rs109055951	24720352	0.286
rs109151890	24724974	0.287
rs135573576	24729765	0.286
rs136860540	24736064	0.145
rs134726969	24737917	0.295
rs109028875	24758139	0.000
rs110334248	24761860	0.106
rs109490367	24763610	0.107
rs110321820	24765731	0.106
rs109783781	24769617	0.107
rs109003440	24772375	0.166
rs109172777	24779419	0.106
rs133640284	24783381	0.051
rs41627956	24787245	0.166
rs134998417	24797724	0.105
rs134309686	24805520	0.104
rs137425866	24808828	0.000
rs133467103	24824037	0.052
rs134623289	24828922	0.048
rs134435992	24864286	0.017
rs136166205	24869608	0.301
rs133846946	24874608	0.287
rs110367762	24892678	0.515
rs133921678	24894527	0.034
rs135626029	24897094	0.034
rs133142988	24900445	0.034
rs134735082	24902136	0.034
rs137627685	24906337	0.034
rs109116062	24909247	0.680
rs137044774	24911824	0.034
rs109110003	24913654	0.188
rs133409868	24915886	0.034
rs134649249	24922753	0.408
rs135388492	24931388	0.409
rs133480234	24933932	0.409
rs134848602	24939285	0.409
rs137780934	24941523	0.034
rs133714277	24944254	0.034
rs137200131	24952035	0.034

rs136544328	24956145	0.034
rs134174250	24958417	0.034
rs135735870	24961879	0.530
rs110243083	24973324	0.265
rs136888475	24975563	0.034
rs135318045	24980786	0.034
rs136631581	24989997	0.034
rs109636480	24998326	0.433
rs135404594	25001051	0.034
rs134286310	25009960	0.035
rs135538206	25012733	0.034
rs109815800	25015640	1.000
rs137204453	25018843	0.034
rs135852767	25021594	0.034
rs136828442	25026174	0.034
rs133840388	25036693	0.034
rs135401045	25050448	0.034
rs136474498	25054377	0.206
rs133704402	25056208	0.206
rs137401639	25058053	0.552
rs136288032	25060055	0.034
rs136982189	25066322	0.438
rs135114806	25069487	0.034
Chr14_25075542	25075542	0.034
rs134048394	25079291	0.034
rs135256588	25082358	0.034
rs136629079	25092241	0.034
rs41722894	25098364	0.145
rs137557469	25102663	0.034
rs133531622	25105265	0.034
rs41627954	25107556	0.117
rs41722912	25111082	0.217
rs41722915	25114769	0.248
rs41722918	25117469	0.214
rs134949970	25119622	0.034
rs41722865	25123059	0.242
rs136826029	25129005	0.388
rs41722872	25134787	0.173
rs135269914	25147967	0.379
rs43151427	25154132	0.062
rs43151429	25160597	0.015
rs135211309	25164603	0.001
rs43157016	25173600	0.015
rs43157018	25174741	0.151
rs43157020	25175950	0.015
rs43157023	25180411	0.126
rs133606272	25185357	0.015
rs41720594	25199512	0.015
rs41720598	25203669	0.002
rs137338872	25204467	0.092
rs41720621	25215027	0.003
rs41720613	25215969	0.003
rs136157418	25221694	0.142
rs41720630	25225097	0.003
rs41721832	25226878	0.003
rs41721841	25228905	0.410
rs41722127	25235472	0.090
rs132881564	25241366	0.169
rs41722847	25249177	0.000
rs134732749	25252182	0.217
rs41722855	25254540	0.090
rs41722039	25267800	0.077
rs136598772	25272140	0.083
rs41722053	25276491	0.345
rs41578094	25284162	0.345
rs137648164	25287012	0.123

rs133403697	25290225	0.074
rs134650233	25293271	0.459
rs135388393	25298972	0.486
rs41627953	25307116	0.626
rs136191791	25315687	0.453
rs41722103	25320421	0.244
rs42892600	25329035	0.621
rs42892592	25332510	0.359
rs42892582	25336906	0.001
rs42892571	25343470	0.001
rs42892565	25348919	0.019
rs42892557	25351733	0.001
rs109227633	25354674	0.019
rs137490412	25358895	0.034
rs132872540	25365895	0.034
rs135577401	25374602	0.034
rs133012258	25376827	0.034
rs134286113	25379505	0.034
rs135530224	25383331	0.034
rs133193436	25389001	0.034
rs134485671	25391747	0.000
rs110816518	25393163	0.368
rs109871644	25396031	0.063
rs109782798	25400645	0.200
rs41657755	25401722	0.371
rs110576056	25412013	0.004
rs110602867	25423298	0.000
rs41722033	25425357	0.000
rs133919865	25429707	0.034
rs109679763	25446793	0.000
rs137844185	25451730	0.034
rs110020650	25457504	0.000
rs41627950	25459674	0.163
rs41721289	25460952	0.163
rs41721294	25466175	0.163
rs134038032	25468481	0.034
rs137536818	25471170	0.047
rs135252751	25474456	0.034
rs109056763	25478810	0.071
rs109216574	25488048	0.406
rs110108793	25490226	0.574
rs110506327	25492467	0.702
rs110741347	25497146	0.575
rs41720428	25498881	0.702
rs132979341	25500235	0.702
rs110632518	25501417	0.126
rs136543212	25502915	0.702
rs41627948	25504073	0.574
rs137267491	25505663	0.574
rs41627946	25506575	0.574
rs136889989	25507730	0.574
rs135262614	25510859	0.574
rs133736127	25513599	0.574
rs41720387	25517111	0.032
rs41720383	25518123	0.032
rs41721322	25520749	0.577
rs109670294	25521888	0.576
rs132786957	25525225	0.577
rs41623108	25526683	0.577
rs136345290	25528516	0.702
rs133597776	25529645	0.574
rs134607819	25530203	0.574
rs136855191	25536019	0.574
rs134846474	25537252	0.574
rs137619218	25541189	0.574
rs133319071	25544079	0.407
rs134551820	25548323	0.068

rs136150430	25551817	0.034
rs133834218	25555197	0.034
rs134633991	25557034	0.034
rs42935363	25567411	0.126
rs135313923	25570551	0.034
rs109633597	25577162	0.084
rs109418171	25579888	0.126
rs43093130	25582834	0.007
rs134725247	25584875	0.025
rs136320412	25586296	0.021
rs109761795	25589517	0.324
rs110985019	25601303	0.329
rs109521494	25608094	0.324
rs29021334	25612510	0.232
rs29021333	25616884	0.237
rs42962539	25621782	0.007
rs108941421	25638580	0.012
rs42961225	25640190	0.069
rs42299113	25648989	0.069
rs137236027	25650993	0.034
rs109540593	25655658	0.015
rs134159539	25659050	0.034
rs42299126	25664934	0.006
rs135316058	25675568	0.051
rs136667611	25683113	0.034
rs134206288	25686207	0.034
rs110774011	25698286	0.030
rs133094347	25699163	0.030
rs134370861	25704807	0.030
rs135531050	25708285	0.030
rs42839864	25715320	0.004
rs42839872	25719951	0.001
rs134649406	25725057	0.033
rs42839876	25730129	0.000
rs42839873	25731992	0.001
rs133249705	25733426	0.001
rs42839886	25739308	0.001
rs109067020	25747953	0.000
rs137397008	25753013	0.033
rs43010099	25759873	0.001
rs43010094	25762795	0.002
rs43010082	25766228	0.016
rs43010073	25767656	0.002
rs43010065	25769988	0.002
rs41665905	25771436	0.002
rs135254559	25776037	0.040
rs43757985	25779560	0.003
rs137551408	25786908	0.033
rs133519399	25794261	0.032
rs43770985	25797331	0.016
rs135744414	25800191	0.033
rs133347432	25803886	0.033
rs134947467	25808557	0.033
rs43770972	25812326	0.001
rs133570825	25814803	0.033
rs43770969	25817300	0.150
rs137748068	25819872	0.033
rs133628406	25823040	0.033
rs137494880	25826189	0.033
rs135939284	25828312	0.033
rs42299100	25832112	0.048
rs133003803	25835618	0.033
rs136976295	25839257	0.033
rs135202659	25842735	0.033
rs42299083	25846511	0.008
rs134006862	25849150	0.000
rs42299080	25851646	0.075

rs41665281	25857110	0.213
rs134601995	25860105	0.000
rs136141080	25863924	0.000
rs133252286	25866853	0.000
rs137336582	25869266	0.039
rs135734725	25871315	0.076
rs136755107	25873843	0.000
rs134567839	25877586	0.003
rs136146069	25882396	0.000
rs41665280	25887784	0.039
rs133829973	25894793	0.000
rs134624153	25899663	0.080
rs42299038	25909536	0.091
rs41665273	25913294	0.093
rs137492335	25915281	0.033
rs132878276	25917492	0.033
rs42298501	25921382	0.032
rs42298505	25926527	0.041
rs133124974	25930713	0.066
rs134858624	25933870	0.032
rs136316897	25938065	0.044
rs42299032	25946432	0.086
rs110721536	25947476	0.084
rs110797611	25956248	0.005
rs42298481	25959516	0.406
rs110342609	25962036	0.242
rs42298477	25964134	0.406
rs109372952	25966829	0.206
rs135375478	25972263	0.032
rs42298471	25979073	0.079
rs109341059	25980137	0.022
rs42298470	25982072	0.079
rs110558178	25983064	0.022
rs110982026	25985624	0.036
rs42298467	25986431	0.237
rs29017100	25987996	0.027
rs29017103	25991165	0.174
rs42306917	25992595	0.079
rs137839813	25999691	0.016
